# Pulmonary hypertension in systemic lupus erythematosus: a systematic review and analysis of 642 cases in Chinese population

**DOI:** 10.1007/s00296-012-2525-y

**Published:** 2012-09-16

**Authors:** Y. K. Xia, S. H. Tu, Y. H. Hu, Y. Wang, Z. Chen, H. T. Day, K. Ross

**Affiliations:** 1 Department of Integrated Chinese and Western Medicine, Tongji Hospital of Tongji Medical College, Central-China (Huazhong) University of Science and Technology, 1095 Jiefang Avenue, Wuhan, 430030 Hubei People’s Republic of China; 2 ODS Health Plans, Portland, OR USA; 3 Oregon State University, Corvallis, OR USA

**Keywords:** Pulmonary hypertension, Systemic lupus erythematosus, Raynaud’s phenomenon, Serous effusion, Right heart catheterization, Prognosis

## Abstract

Pulmonary hypertension (PH) is an increasingly recognized complication of systemic lupus erythematosus (SLE). To develop a more comprehensive understanding of the clinical and pathological characteristics of pulmonary hypertension associated with systemic lupus erythematosus (PH/SLE) in the Chinese population, a systematic review of the literature up to 2012 was conducted. Six hundred and forty-two Chinese PH/SLE cases from 22 studies were identified as well documented and further analyzed. Transthoracic echocardiography (TTE), X-ray, electrocardiogram and right heart catheterization (RHC) were performed to diagnose PH in SLE patients. The mean age of subjects was 35.5 years, the male to female ratio was 1:14, and the mean duration of SLE when PH was diagnosed was 10.7 years. The prevalence of PH in SLE was 2.8–23.3 %. Symptoms were usually nonspecific, and the observed clinical characteristics include Raynaud’s phenomenon (41.4 %), serous effusion (27.7 %), positive RNP (51.5 %) and positive ACL (46.6 %). Gold standard RHC is strongly recommended, especially for those who had resting pulmonary arterial systolic pressure >30 mmHg on TTE with the aforementioned clinical characteristics. Corticosteroids, immunosuppressants and vasodilators were the most common medications employed in treatment. Early identification and standard PH treatment with intensive SLE treatment can improve the prognosis.

## Introduction

Pulmonary hypertension (PH) is a severe, potentially life-threatening complication of systemic lupus erythematosus (SLE) [1, 2]. The reported prevalence of PH in SLE ranged from 0.5 to 14 % in a previous review of the literature [3]. The mechanisms involved in the pathogenesis of PH are largely unknown. Elevated antiendothelial cell antibodies (aECA) were detected and reported in patients with SLE, especially with PH, which implied that aECA may be involved in the pathogenesis of vascular injury [4]. The association of PH with SLE suggests an underlying inflammatory component in the pathogenesis of this syndrome [5].

In SLE, the prognosis of patients with PH has been reported to be very poor and the mean survival from the onset of PH was 2 years [6–9]. PH is the third most common cause of death in SLE following infection and organ failure [10]. With the widespread availability of high-quality imaging systems and a better understanding of PH pathology [11, 12], the number of such patients reported in the Chinese literature has been steadily increasing. To better understand PH in SLE and to raise more awareness and help clinicians, a systematic review of the literature up to 2012 was conducted; clinical and pathological characteristics of PH associated with SLE were examined; well-documented Chinese cases were identified and further analyzed with the aim to provide a more comprehensive understanding of PH in SLE in the Chinese population.

## Methods and materials

A comprehensive literature search was conducted to retrieve up-to-date PH in SLE-related information up to 2012 from multiple sources including the Chinese Biology and Medicine Database (CBMD), China Hospital Knowledge Database (CHKD), Wanfang Database, VIP Information Database, PubMed, Embase, Web of Science and Ovid. Indexing keywords used for the search and retrieval included PH, PAH, PHT, secondary pulmonary hypertension, primary pulmonary hypertension, lupus and systemic lupus erythematosus.

With a clear focus on PH in SLE in the Chinese population, all retrieved articles were carefully reviewed and checked for possible duplication to avoid the repetitive adoption of the same data and materials. The inclusion criteria were made that SLE diagnosis has to meet at least the 1982 revised ACR criteria of SLE [13] and diagnosis of PH was made when the resting pulmonary arterial systolic pressure (PASP) was >30 mmHg at rest by TTE (transthoracic echocardiography). Excluded were other forms of PH due to left heart disease, chronic lung disease and/or hypoxemia, chronic thromboembolism and a miscellaneous group as well as PH associated with other autoimmune diseases (such as systemic sclerosis, mixed connective tissue disease and so on).

All unduplicated cases meeting the above inclusion and exclusion criteria were entered into a database. Cases were considered well documented if they have at least 5 of the following 7 categories of information: age, gender, symptoms, imaging, laboratory tests, treatment data (from the clinical records) and follow-up. A total of 22 studies with 642 well-documented PH–SLE cases were included and analyzed. All statistical analyses were performed using SPSS 11.0 for Windows.

## Results

Twenty-two studies with 642 well-documented PH–SLE cases were included in this analysis. Thirteen studies (59.1 %) were from Beijing, Shanghai and Guangzhou (the capital of Guangdong province, adjacent to Hong Kong). A sum of 430 patients came from these three cities, representing two-thirds (67 %) of the total 642 cases. Information about patients meeting the well-documented SLE–PH criteria is listed in Table 1.

**Table 1:** Patients meeting the well-documented SLE–PAH criteria

Criteria	*n*	%
Age (years)	642	100.0
Gender	642	100.0
Symptoms	466	72.6
Imaging	642	100.0
Laboratory tests	532	82.9
Treatment	273	42.5
Follow-up	266	41.4

Total *n* = 642

Of the 642 patients, 42 were males and 600 were females. The male to female ratio was 1:14. Their age ranged from 8 to 85 years (with a mean age of 35.5), and their disease duration ranged from 0.5 to 50.5 years (with a mean of 10.7 years). The prevalence rate of PH in SLE reported in 12 studies ranged from 2.8 to 23.3 %.

The symptoms were usually nonspecific, and often coexisted with several clinical characteristics. The three most commonly observed were Raynaud’s phenomenon, arthritis and serous effusion (Table 2).

**Table 2:** Symptoms of PAH associated with SLE patients (*n* = 466)

Symptoms	Patients (*n*)	%
Raynaud’s phenomenon	193	41.4
Arthritis	178	38.2
Serous effusion	129	27.7
Serositis	101	21.7
Pulmonary fibrosis	83	17.8
Polypnea	58	12.4
Fatigue	57	12.2
Rash	49	10.5
Acra vasculitis	37	7.9
Fever	29	6.2
Oral ulcer	24	5.2
Alopecie	20	4.3
Chest distress	16	3.4
P2 accentuation	15	3.2
Butterfly erythema	12	2.6
Photosensitivity	11	2.4
Palpitation	10	2.1
Dry mouth and eye	9	1.9
Limbs edema	9	1.9
Dyspnea	6	1.3
Myosalgia	5	1.1

One hundred and ninety-three patients (41.4 %) from 17 studies had Raynaud’s phenomenon. In seven studies, 51.2 % (32.4–77.8 %) of lupus patients with PH had Raynaud’s phenomenon versus 19.9 % (10.3–36 %) of lupus patients without PH. They reported correlation of Raynaud’s phenomenon with PH.

One hundred and seventy-eight patients (38.2 %) from 12 studies had arthritis, mostly proximal interphalangeal arthritis. Lupus is sometimes misdiagnosed as rheumatoid arthritis because arthritis may precede other system damages.

One hundred and twenty-nine patients (27.7 %) from eight studies had serous effusion—primarily pericardial effusion (88) and pleural effusion (10). In four studies, 57.3 % (44.4–63.9 %) of lupus patients with PH had serous effusion versus 21.7 % (15.2–25 %) of lupus patients without PH. They reported correlation of serous effusion with PH.

One hundred and one patients (21.7 %) from six studies had serositis. In four studies, 60.5 % (53.7–64.9 %) of lupus patients with PH had serositis versus 29 % (25.6–32.9 %) of lupus patients without PH. They reported correlation of serositis with PH.

TTE, electrocardiography, chest X-ray and right heart catheterization (RHC) were performed for the purpose of diagnosing PH in lupus patients. The pulmonary artery pressures of all 642 patients were measured by TTE. Two studies compared the sensitivity of TTE with chest X-ray and electrocardiography and reported the detection rates as 93.3, 26.7 and 26.7 %, respectively.

Among all the laboratory findings, positive ANA, positive ACL and positive RNP were significantly higher in the lupus patients with PH. As seen in Table 3, the anti-ACL antibody in 198 patients (46.6 %) from 14 studies was positive. In five studies, 51.3 % (46.2–56.8 %) of lupus patients with PH had positive anti-ACL antibody versus 23.8 % (16–28.9 %) of lupus patients without PH. They all reported positive correlation of the presence of anti-ACL antibody with the diagnosis of PH on TTE.

**Table 3:** Auto-antibodies of SLE patients with PAH

Auto-antibodies	Patients	Positivity, *n* (%)
ANA (+)	230	222 (96.5)
Ds-DNA (+)	283	167 (59.0)
RNP (+)	334	172 (51.5)
SSA (+)	271	127 (46.9)
ACL (+)	425	198 (46.6)
Rib (+)	9	3 (33.3)
Antithrombase (+)	72	24 (33.3)
SSB (+)	245	73 (29.8)
Antiplasmin (+)	72	21 (29.2)
Sm (+)	257	68 (26.5)
β2-GPI (+)	72	18 (25.0)
AHA (+)	62	15 (24.2)
ANCA (+)	139	18 (12.9)
Scl-70 (+)	47	6 (12.8)
Anti-APC (+)	72	8 (11.1)
Jo-1 (+)	15	1 (6.7)
T-PA (+)	72	3 (4.2)

*ANA* anti-nuclear antibody, *Anti-ds-DNA* anti-double-stranded DNA, *RNP* anti-ribonucleoprotein antibody, *SSA* anti-Sjogren syndrome A antibody, *ACL* anti-cardiolipin antibody, *Rib* anti-ribosome antibody, *SSB* anti-Sjogren syndrome B antibody, *Sm* anti-Smith antigen, *β2-GPI* anti-cardiolipin-β2-glycoprotein I complex, *AHA* anti-histone antibody, *ANCA* anti-neutrophil cytoplasmic antibody, *Scl-70* anti-topoisomerase 1 antibody, *APC* anti-activated protein C antibody, *Jo-1* anti-histidyl-tRNA synthetase antibody, *T-PA* anti-tissue-type plasminogen activator antibody

The anti-RNP antibody in 172 patients (51.5 %) from twelve studies was positive. In five studies, 49.2 % (13–80.5 %) of lupus patients with PH had positive anti-RNP antibody versus 20 % (3.8–37.7 %) of lupus patients without PH. They all reported positive correlation of the presence of anti-RNP antibody with the diagnosis of PH on TTE.

The anti-β2-GPI antibody in 72 patients (25 %) from one study was positive; 25 % of lupus patients with PH had positive anti-β2-GPI antibody versus 12 % of lupus patients without PH. And it reported positive correlation of the presence of anti-β2-GPI antibody with the diagnosis of PH on TTE.

Corticosteroids, immunosuppressants, vasodilators and anticoagulants were the commonly used medications for the patients in this analysis (Table 4).

**Table 4:** Medicine treatment for 273 patients

Medicines	Patients	%
Corticosteroids	268	98.2
Immunosuppressant	179	65.6
Vasodilators	143	52.4
Anticoagulants	9	3.3
Other medicines	23	8.4

All 273 patients (42.5 %) from ten studies took two kinds of corticosteroids as basic treatment—oral administration (87.2 %) and intravenous injection (11 %). Cyclophosphamide was used in 156 patients (57.1 %) from the same ten studies.

Two hundred and sixty-six patients (41.4 %) from nine studies were followed up for 0.75–12 years (with a mean of 3.8 years). Forty-one of them (15.4 %) with PH died during the follow-up period. Only six studies reported the causes of death, which included right heart failure, kidney failure, severe infection, pulmonary embolism, cardiac arrest, arrhythmia, alimentary tract hemorrhage, cerebral vascular accident and sudden death. One hundred and thirteen patients (42.5 %) showed 23.8 % (4.5–73.3 %) improvement of their right ventricular systolic pressure by Doppler echocardiography after appropriate drug intervention. Sixty-eight patients (25.6 %) showed 11.6 % (1.8–61.7 %) aggravation of PH after treatment.

## Discussion

Pulmonary hypertension (PH) has been reported in almost all rheumatic diseases including systemic sclerosis and SLE [14–17]. It is an increasingly recognized manifestation of SLE and an important reason for morbidity and mortality [18, 19]. SLE with PH attracts more and more clinical and academic attentions.

It takes time for SLE–PH to be recognized and reported. In 1954, Harvey et al. [20] reported no PH in 138 SLE patients. In 1981, Perez and Kramer reported a 9 % prevalence rate of PH in SLE patients [21]. The reported PH prevalence rates varied greatly depending on the type of study. Retrospective studies reported prevalence rates of 0.5–6 % [19], while prospective studies in asymptomatic patients found higher prevalence rates of 9–14 % [6, 22]. In this analysis, we found the prevalence rate of SLE–PAH in China ranged from 2.8 to 23.3 % as reported by twelve retrospective studies. The wide variability in the reported prevalence rates reflects the varying definitions of PH, differences in diagnostic methods, population groups studied and number of patients involved [23]. The prevalence rate from prospective cross-sectional cohort study would be better than the retrospective smaller studies, but no reports were found yet.

It is noteworthy that more than half of patients in this analysis originated from the three big metropolitan cities (Beijing, Shanghai and Guangzhou) in China. This may not necessarily indicate that the prevalence of SLE–PH in the three places was higher than the rest of China. It may simply be that these cities have more medical resources and expertise in recognizing and diagnosing PH and patients there can better afford the costs of medical care.

The pathogenesis of PH in SLE is not well understood. Abnormal vasospasm, Raynaud’s phenomenon, platelet abnormalities, vasculitis, thrombosis and thromboembolism have been suggested as possible pathogenetic factors [8, 24, 25]. Endothelial dysfunction is strongly implicated in the pathogenesis of autoimmune vasculitic diseases including lupus [26, 27]. Nakamura et al. [28] found the elevation of endothelin may be associated with PH and possibly related to disease activity in a murine lupus model. Shen et al. [29] reported that PH patients had higher serum endothelin more commonly in the active stage and the level of endothelin was correlated with pulmonary pressure. In addition, a variety of auto-antibodies in vivo of lupus patients can cause vascular endothelial cell injury such as ACL, LA, ANA, ds-DNA. They could directly damage the endothelial cells or form immune complexes and then deposit in the vascular wall, leading to vasoconstriction, platelet aggregation and the formation of thrombosis. In our study, we found that positive ACL and positive RNP were correlated with PH and they were strong predictors of PH in some studies, which are compatible with previous findings [30–32]. Only one study reported 25 % anti-β2-GPI antibody was positive in 72 SLE–PH patients, which is consistent with Houman’s research studied in Tunisia (25 % of positive for anti-β2-GPI in 100 patients) [33].

Most cases of SLE-associated PH are mild, which makes early diagnosis difficult, and only those cases presenting with severe PH have been reported. Lian et al. [30] suggested that more than 40 % of the SLE–PH patients would not have observable symptoms when early PH persisted. In general, the clinical symptoms of SLE patients with PH are nonspecific such as progressively exertional dyspnea, chest pain, nonproductive cough, edema, easy fatigue, impaired exercise tolerance [14], and can also be caused by many other factors including pleural or pericardial effusions, interstitial lung disease, making it possible to miss the diagnosis [34]. Physical findings include a loud second pulmonic heart sound, pedal edema, serous effusion, Raynaud’s phenomenon and so on. In this study, the nonspecific symptoms reported above were not so common as Raynaud’s phenomenon and serous effusion. One hundred and ninety-three patients (41.4 %) presented Raynaud’s phenomenon. Raynaud’s phenomenon is characterized by vasospasm and could be the cutaneous manifestation of general vascular disorder resulting in pulmonary arterial vasoconstriction [3, 35]. The high frequency of Raynaud’s phenomenon patients with SLE and PH suggests a potential role for etiology of PH, and also supports the notion that Raynaud’s phenomenon is a pathogenic risk factor and a marker for disease severity [30, 36].

TTE has been widely used to screen for PH due to its safety, convenience and sensitivity. Denton et al. [37] reported that TTE had a sensitivity of 90 % and a specificity of 75 % compared with RHC. Gonzalez et al. [38] compared TTE with RHC to detect PH and found that the method of applying differential pressure of tricuspid regurgitation for estimating pulmonary hypertension had high accuracy with a sensitivity of 83.3–90 % and a specificity of 75 %. RHC is considered the gold standard test in the diagnosis and classification of PH. PH may be caused by venous or arterial changes, and pulmonary vasculitis in SLE patients can lead to pulmonary arterial hypertension (PAH). RHC is currently the only way to differentiate what kind of PH that SLE patients suffered from. Besides, with an assessment of vasoreactivity, RHC is necessary if PH is highly suspected and is needed for decision making with particular respect to treatment. Jais et al. [39] emphasized that RHC was mandatory before starting any treatment and for following-up PAH patients and showed it provided strong predictors of response to immunosuppressive therapy by reliably measuring cardiac index and calculating pulmonary vascular resistance.

Insidious onset of SLE-associated PH develops rapidly. It is irreversible and usually of poor outcome with a 2- to 5-year survival after initial diagnosis [40]. Therefore, early, intensive treatment for the primary disease (SLE) is the key to improve prognosis. Various drugs have been used for the treatment of SLE-associated PH, such as corticosteroids, immunosuppressants, vasodilators, endothelin receptor antagonists and anticoagulants. They could improve the symptoms of lupus, and only some of them had an effect on decreasing the pulmonary artery pressure. But there was a lack of detailed comparison of the results of different combined therapies. Corticosteroids plus immunosuppressants can block the immune-mediated vascular inflammation in the early stage. Promising results have been obtained with combination therapy that includes steroids and cyclophosphamide in patients with early stages of PH [22]. Abnormal pulmonary and cardiac function can be improved by immunosuppressants and corticosteroids alone or combined with pulmonary vasodilators [39]. If a patient is strongly vasodilator responsive, then treatment with high dosage of calcium channel blocker should be adequate [34]. It has been proved that traditional vasodilators could improve hemodynamics, increase exercise tolerance and prolong survival time by reducing right ventricular afterload. However, there are potential undesirable response such as an increase in ventilation/perfusion mismatch and a reduction in systemic vascular resistance, leading to hypotension [20, 41]. If a patient is not vasodilator responsive, then treatment would include endothelin receptor blockers [34]. There are two kinds of endothelin receptor blockers: selective and nonselective. Bosentan, a nonselective endothelin receptor blocker, was reported by Ahmadi-Simab et al. [42] to have a good effect on relieving PH. Mok et al. [43] found 12 months of bosentan treatment resulted in significant improvement in six-minute walk distance (6MWD), transient or sustained drop in systolic PAP. Because of endothelial dysfunction in lupus patients with PH, long-term hypoxia and hypercapnia lead to secondary polycythemia, hypercoagulability, stasis of the microcirculation, and then increasing risk of thrombosis and embolism. Thus, anticoagulant therapy has been recommended to be used with other medicines such as corticosteroids and immunosuppressants. However, the effect of warfarin anticoagulation is controversial for the treatment of idiopathic PAH patients, and there is no evidence to support that anticoagulant therapy provides benefit for SLE–PAH patients.

Some Chinese herbals may also relieve PH. Tang et al. [44] found ligustrazine extracted from ligustici could decrease the level of endothelin through inhibiting the expression and release of ET-1 in hypoxic PH rats. Jiang et al. [45] observed salvia miltiorrhiza may depress the secretion of endothelin and then reduce hypoxic pulmonary vasoconstriction. Gene therapies for PH have been successful in the laboratory with the potential for effective treatments. Lung transplantation is the only measure to treat advanced PH, and 1-year survival rate after transplantation is 65–70 %.

In general, the prognosis in mild and moderate disease is not certain, depends on different therapies and courses of treatment. However, SLE patients with severe symptomatic PH carry a poor prognosis. Oren et al. [46] reported the overall 2-year survival was ≤50 % when pulmonary hypertension was present. Chung et al. [47] reported PH in SLE untreated had a 3-year mortality of 54.1 %. In recent years, SLE patients have improved survival rate closer to idiopathic PAH with the advent of PAH-specific therapies [48, 49].

A major limitation of this study is that RHC confirmation of PH was not performed on all patients. PASP > 30 mmHg on TTE in lupus patients was chosen a priori, and there are concerns about TTE’s sensitivity and specificity. In addition, most studies were retrospective with small sample sizes. Therefore, multi-center prospective cohort studies of SLE patients with PH are called for in China.

In conclusion, PH is an increasingly recognized complication in SLE. PH associated with SLE typically affects middle-age women and is often characterized by nonspecific symptoms and poor prognosis. Early diagnosis is essential for SLE patients with PH. Gold standard RHC is strongly recommended since symptoms of PH in lupus patients are nonspecific, especially for those who had PASP > 30 mmHg on TTE with positive anti-ACL or anti-RNP antibodies or Raynaud’s phenomenon or serous effusion or serositis. Early identification, standard PH treatment as well as intensive treatment for SLE can improve the prognosis.
